# Success and Complication Rates of Transvenous Lead Extraction in a Developing High-Volume Extraction Center: The Zurich Experience

**DOI:** 10.3390/jcm12062260

**Published:** 2023-03-14

**Authors:** Daniel Hofer, Noah Kuster, Michelle C. Bebié, Tom Sasse, Jan Steffel, Alexander Breitenstein

**Affiliations:** Division of Electrophysiology, Department of Cardiology, University Heart Center, University Hospital Zurich, 8091 Zurich, Switzerland

**Keywords:** lead extraction, success, complication, single center, outcome

## Abstract

Introduction: Transvenous lead extractions are increasingly performed for malfunction or infection of cardiac implantable electronic devices, but they harvest a potential for complications and suboptimal success. Apart from multicenter registries and reports from highly experienced single centers, the outcome in individual newly developing high-volume centers starting a lead extraction program is less well established. We aimed to evaluate the clinical and radiological success and complication rate at our center, having started a lead extraction program less than a decade ago. Methods: We retrospectively analyzed patients who underwent transvenous lead extraction at the University Hospital Zurich from 2013 to 2021 regarding success as well as complications and compared our results to previously reported outcome rates. Results: A total of 346 patients underwent 350 transvenous lead extractions from January 2013 to December 2021. Combined radiological success was achieved in 97.7% and clinical success in 96.0% of interventions. Procedure-related major complications occurred in 13 patients (3.7%). Death within 30 days after transvenous lead extractions occurred in 13 patients (3.7%), with a procedure-related mortality of 1.4% (five patients). Summary: Transvenous lead extractions in newly developing high-volume centers can be performed with high clinical and radiological success rates, but procedure-related major complications may affect a relevant number of patients. Compared to large single or multicenter registries of experienced centers, the success rate may be lower and the complication rate higher in centers newly starting with lead extraction, which may have important implications for patient selection, procedural planning, proctoring, and safety measures.

## 1. Introduction

Since the introduction of transvenous cardiac pacing in the past century, the number of implantations of cardiac implantable electronic devices (CIED) has dramatically increased over the years, with by now approximately 1,000,000 de novo implanted transvenous leads every year worldwide [1]. The increase in implantation can largely be explained by population growth, longer life expectancy, facilitated healthcare access, and expanding indications for device therapy [2]. Because of lead dysfunctions or device infections, the number of transvenous lead extractions (TLEs) performed has also increased, with an estimated 10,000–15,000 device leads being extracted every year worldwide using specialized tools [3]. TLE techniques have been steadily improved to increase efficiency and safety, but the population in need of TLE is also becoming older and often demonstrates more comorbidities, more complex device systems, and more prior device interventions [4]. Previously available outcome rates of TLE are mainly derived from large single or multicenter registries of highly experienced extraction centers with a reported clinical success rate of 96.7 to 97.7, an overall combined radiological success rate of 93 to 99.3%, and a reported major procedure-related complication rate of 0.4 to 2.3% [5,6,7,8,9]. However, the outcome after TLE may differ in the real-world setting outside of highly experienced centers and the associated registries, with limited experienced operators, and in only just developing high-volume centers [10,11]. Our center started with a supervised and structured lead extraction program in 2013 and surpassed 30 TLEs per year as a standard for a high-volume center in 2018 with 51 TLEs, resulting in 72 TLEs in 2021 [7,12]. For this study, we retrospectively analyzed the success and complication rates of TLE in our developing high-volume center in the last 8 years, and compared the outcome to larger registries of centers with longer high-volume experience. 

## 2. Materials and Methods

### 2.1. Study Design

We retrospectively analyzed all patients undergoing TLE at the university hospital Zurich from the beginning of 2013 (reflecting the start of a standardized and supervised extraction program) until the end of 2021. Every TLE procedure was considered as a separate entity, without regard to patient identity, in order to allow for multiple TLE within the same patient to be included in this study, as long as the second TLE procedure was not performed during the same hospital stay. Patients undergoing lead explantation (see Section 2.4 Definitions) were purposely excluded, as were patients undergoing lead extraction during open chest surgical approaches. Because of the retrospective design, the choice of extraction tools and their size was always left at the discretion of the operator. This study was approved by the local ethics committee (BASEC-NR: 2018-01540).

### 2.2. Extraction Procedure

**Figure 1:**
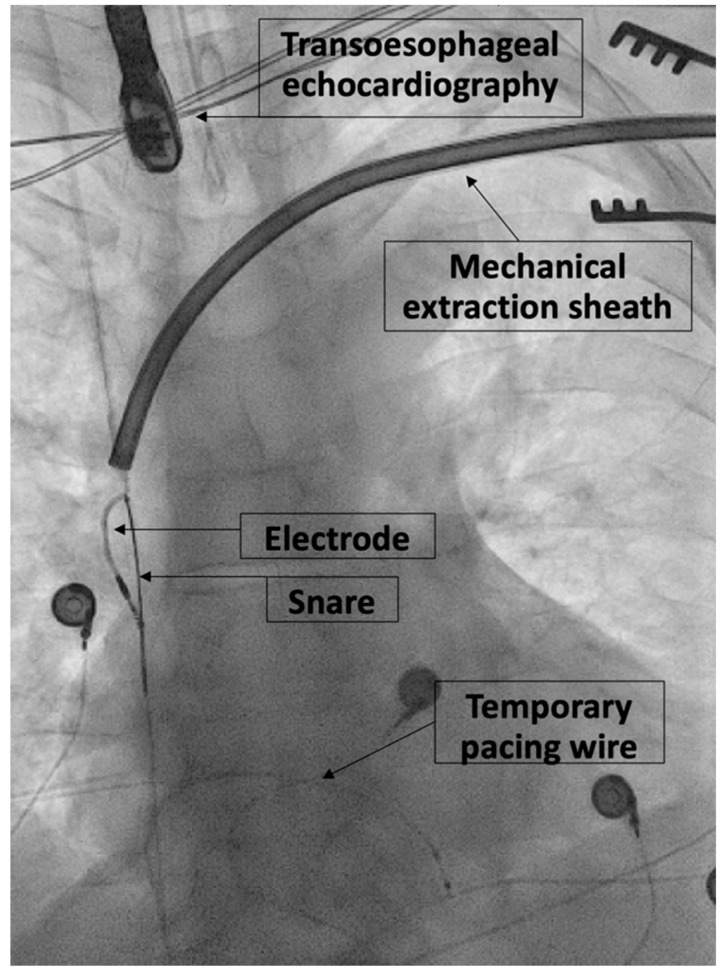
X-ray during lead extraction with combined superior and femoral approach.

Participating physicians were a cardiac surgeon at the beginning of the study period and three cardiologists with electrophysiology (EP) specialty in the latter part of the study period. TLE was performed under general anesthesia or conscious sedation based on the decision of the operator. TLE was performed in a hybrid operating room in the majority of procedures, and a standard electrophysiology laboratory in a minority of procedures. A superior approach via the implant-related vein was always the primary TLE method unless this approach had previously failed. If simple traction failed, a locking stylet (Liberator^®^ Beacon^®^ Tip Locking Stylet, Cook Medical, Bloomington, IN, USA) was introduced into the lumen of the lead and deployed. To increase lead control, a compression coil (One-Tie^®^ Compression Coil, Cook Medical) was used. Because of historical extensive in-house experience with mechanical rotational lead extraction tools and concerns about safety with laser extraction tools, only mechanical rotational lead extraction tools (TightRail™ and TightRail Sub-C™ by Philips, Amsterdam, The Netherlands, or Evolution^®^ RL and Evolution^®^ Shortie RL by Cook Medical) were used during the study period. If the superior approach failed or additional stability during mechanical lead extraction was needed, a femoral approach using snares (i.e., Needle’s Eye Snare^®^, Cook Medical, or Amplatzer Goose Neck™, Medtronic, Dublin, Ireland) was performed. Some leads were removed using a combination of superior and femoral extraction approaches, especially if insufficient stability or traction during mechanical TLE was suspected (Figure 1).

### 2.3. Data Collection and Analysis

Patient cases were each individually assessed for evaluation of success, failure, and complications. Data collection was performed by studying operation reports, discharge reports, and chest X-rays. Continuous variables are expressed as mean ± standard deviation, while categorical variables are expressed as absolute numbers and percentages. All patient data were anonymized.

### 2.4. Definitions

Definitions were aimed to be as equivalent as possible to the 2009 and 2017 HRS expert consensus and the 2018 EHRA expert consensus statement on lead extraction as well as similar to definitions used in the large multicenter registries to allow comparison (Table 1) [7,12,13,14]. However, due to incomplete annotation in patient reports, some alterations were made in comparison with these expert consensus statements and large multicenter registries: Firstly, we did not differentiate between post-procedural or intra-procedural complications because of prominent lack of data availability in patient charts. Secondly, because ascription of complications may be challenging as patients may undergo additional procedures during their hospital stay, we decided to report all complications identified in medical charts during the hospital stay for TLE. Similar to the EHRA and HRS expert consensus statements, clinical success of TLE was possible in the absence of radiological success, because clinical success and failure of TLE mainly depend on the indication of TLE. In case of infection, only complete CIED removal should be considered a clinical success. In case of indications other than infection, clinical success may be granted despite a remnant of <4 cm, if the remaining lead presumably does not increase the risk of complications (i.e., vascular obstruction) and in the absence of procedure-related major complications [13]. Death after TLE was reported for up to 30 days after TLE in this study. Hereinafter we present our definitions for this study concerning the TLE procedure, success, failure, and complications.

## 3. Results

A total of 346 patients underwent transvenous extraction of 658 leads during 350 interventions from January 2013 to December 2021 and a mean follow-up time of 131.7 months. In four patients, TLE was performed twice during the study period (three for recurring lead defects and one for additional extraction with a jugular snare approach). TLE was performed by four operators during the study period, with one single operator performing 71.4% of all procedures. The mean age at the time of TLE was 63.6 ± 15.3 years, 71.7% of the patients were male, and the mean left ventricular ejection fraction (LVEF) was 45.7 ± 15.1% (Table 2). The main indication for TLE was lead dysfunction (50.3%), followed by infection (30.9%) and device upgrades (10.6%). The mean number of leads extracted per intervention was 1.9 ± 0.8, and the mean lead indwelling time was 112.5 ± 78.6 months (longest 392 months and shortest 13 months). 

### 3.1. Radiological and Clinical Success 

**Figure 2:**
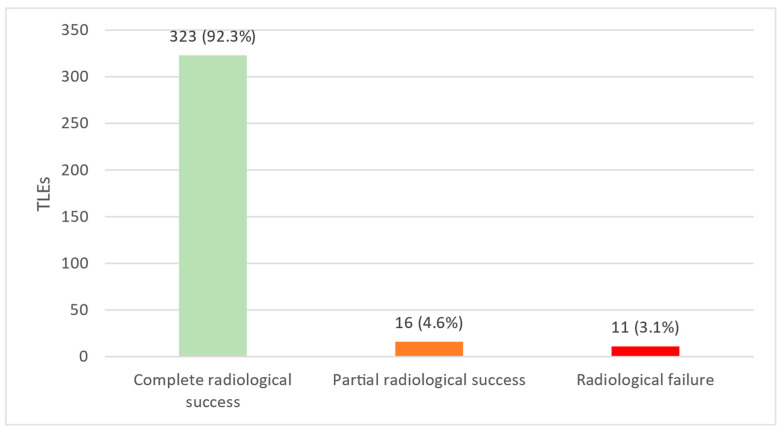
Radiological success or failure following transvenous lead extraction. Out of 350 TLE performed (100%), 323 TLE (92.3%) resulted in complete radiological success, 16 TLE (4.6%) resulted in partial radiological success, and 11 TLE (3.1%) resulted in radiological failure. TLE: transvenous lead extraction.

A total of 323 out of 350 TLE (92.3%) resulted in complete radiological success, and 16 TLE (4.6%) resulted in partial radiological success, cumulating in 96.8% of all TLE, resulting in combined radiological success. A number of 11 TLE procedures (3.1%) resulted in radiological failure (Figure 2). Clinical success was achieved in 329 out of 350 TLEs (94.0%), while 21 TLEs (6.0%) resulted in clinical failure (death within 30 days in 8 TLE, permanently disabling complications in 3 TLE, insufficiently extracted leads in 11 TLE, permanently disabling complications and insufficiently extracted leads in one patient). 

### 3.2. Complications

**Figure 3:**
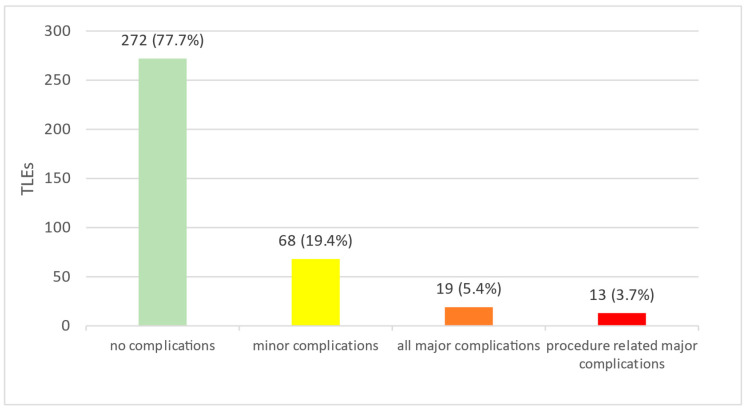
Complications following transvenous lead extraction. Out of 350 TLE performed (100%), 272 TLEs (77.7%) went flawlessly. A total of 68 TLEs (19.4%) resulted in minor complications, 19 TLEs (5.4%) resulted in major complications, and a subgroup of 13 TLEs (3.7%) resulted in major procedure-related complications. One procedure may be listed in multiple groups. TLE: transvenous lead extraction.

Complications occurred in 78 of 350 TLEs (22.3%), of which 68 (19.4%) were minor, 19 (5.4%) were major, and a subgroup of 13 (3.7%) were procedure-related major complications (Figure 3). Reoperations due to complications had to be performed in 20 TLE (5.7%).

### 3.3. Major Complications

**Figure 4:**
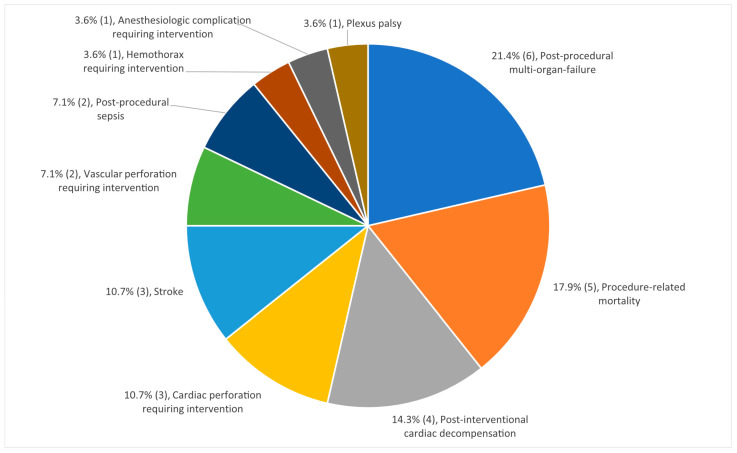
Frequency of major complications. Absolute number in relation to 350 TLEs in brackets. TLE: transvenous lead extraction.

**Figure 5:**
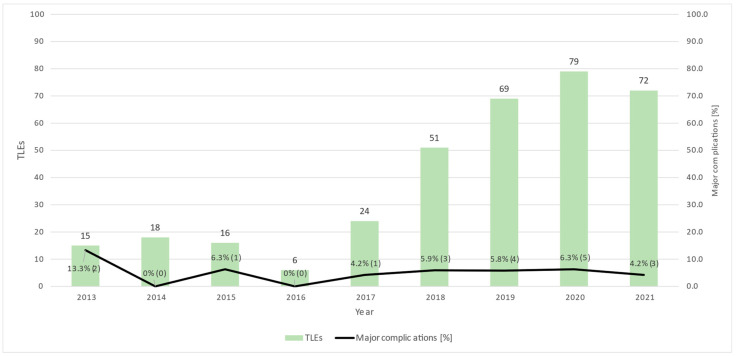
Ratio between TLEs performed and major complications by year. There was an overall increase in TLEs performed from 2013 to 2021. However, the absolute number of major complications was stable, with a subsequent decrease in relative risk. TLE: transvenous lead extraction.

A total of 28 major complications occurred in 19 TLEs (5.4% of all 350 TLEs); the complications are divided into 14 different types (Table 3). The most common major complication was post-procedural multi-organ failure occurring after six TLEs (1.7% of all TLEs, 21.4% of all major complications): five patients developed multi-organ failure after TLE for infective endocarditis and concomitant surgical interventions and one patient developed multi-organ failure after hemorrhagic shock because of TLE-associated hemorrhage. Procedure-related mortality occurred after five TLEs (1.4% of all TLEs, 17.9% of all major complications). Post-interventional cardiac decompensation, cardiac perforation requiring interventions, and stroke followed thereafter (Figure 4). One patient required acute stenting of the vena cava superior for covered rupture of the vena cava superior during TLE, but no sternotomy had to be performed throughout the study period. While the amount of TLE/year increased over time, the yearly rate of major complications remained relatively stable at around 4-6% (Figure 5).

### 3.4. Procedure-Related Mortality

A total of 13 patients (3.7%) died within 30 days after TLE. However, only five deaths were related to the TLE procedure, resulting in a procedure-related mortality of 1.4% (Table 4). Another six patients died before discharge, and two patients passed away outside of the hospital within 30 days (in-hospital mortality of 3.1%). Out of the five presumably procedure-related deaths, two patients died due to a stroke, two patients because of post-interventional cardiogenic shock, and one patient due to post-interventional embolic distal aortic occlusion.

### 3.5. Minor Complications

A total of 82 minor complications occurred in 68 of 350 TLEs (19.4%). The most common minor complication was hematoma without any necessity of intervention (5.1%), followed by newly developed severe tricuspid regurgitation and swelling of the arm/thrombosis (each 2.9%), pericardial effusion without the need for intervention and blood transfusion (each 2.3%), and others (Table 5). 

## 4. Discussion

Our retrospective analysis from a developing high-volume TLE center demonstrated a combined radiological success rate of 96.9% (complete and partial radiological success) and a complete radiological success rate of 92.3%. In comparison, the recent large single or multicenter registries demonstrated slightly higher rates of combined radiological success rate ranging from 98.5 to 99.3%, and complete radiological success ranging from 94.8 to 96.5% [5,6,7,15,16,17]. While there are also studies with lower radiological success rates, different definitions of success and failure complicate comparison [8,16]. Similarly, our clinical success rate of 94.0% was lower than reported success rates from larger established high-volume centers with 96.7–98.7% [5,7,15,16,17]. Regarding complications, we found a slightly higher rate of procedure-related major complications (3.7%) and a higher rate of minor complications (19.4%) compared to reported rates from most large registries (procedure-related major complications 0.4–2.6%, minor complications 1.4–7.2%) [5,6,7,8,9,15,16,17,18,19]. While there are also studies with a higher rate of procedure-related major complications, these often concern specific leads with known increased extraction difficulty [20]. Our rates of procedure-related (1.4%) and in-hospital mortality (3.1%) also appear to be slightly higher than reported mortality rates of experienced and established high-volume centers [5,7,15,17]. For specific complications, we also found post-procedural multi-organ failure and post-interventional decompensation as prominent major complications as opposed to primarily cardiovascular lesions in previous studies, while hematoma after TLE remained the most prominent minor complication, similar to previous studies [5,7,19]. 

In summary, our rates of radiological and clinical success were lower, and complication or mortality rates were higher than previously reported from experienced and established high-volume centers. We only reached the definition of a “high volume TLE center” with more than 30 TLE/year in the last 4 years of this study, and lower TLE experience has previously been shown to be a predictor of mortality and, therefore, procedure failure in TLE, so this may partially explain our results [21]. However, the rate of major complications per year remained relatively stable throughout the study period (Figure 5). Apart from less experience, differences in the definition of success and failure as well as patient and lead characteristics may provide alternative explanations for these differences: while mean patient age was similar in most studies (range from 62.866 years [5,7,15,17,18,19]), our patient population seemed to suffer more frequently from renal insufficiency (34.6% versus 17.4% [7]), potentially suggesting a population with more comorbidities overall. However, comorbidities and medication are sparsely represented in most registries [6,7,18], impeding coherent comparisons with our patient population. Concerning lead characteristics, the number of leads extracted per intervention, presence of ICD leads, and ratio of single/dual-coil ICD leads were similar to previous reports [5,6,7,15,18]. However, our leads demonstrated a definitely longer mean indwelling time of 9.4 years (112.5 ± 78.6 months) compared to the patient population of most previous larger registries with a range of 5.7 to 6.8 years [5,6,7,8,15,18,19]. This may be an important difference in the interpretation of our results, since longer lead dwelling time is a predictor of clinical and radiological failure, as well as complications [5,8]. Additionally, methods concerning the definition of complications and the timeframe of annotated complications vary between registries and publications [5,6,7,8,9,15,16,17,18,19]; we reported all complications during the hospital stay without adjudication in relation to the TLE procedure, which may also partially explain our higher rate of major and minor complications compared to previous publications. 

## 5. Conclusions

Transvenous lead extractions can be performed with high clinical and radiological success rates, but procedure-related major complications may affect a relevant number of patients. Compared to large single or multicenter registries of experienced centers, the success rate may be lower and the complication rate higher in centers newly starting with lead extraction, which may have important implications for patient selection, procedural planning, proctoring, and safety measures.

## 6. Limitations of This Study

The single-center and retrospective design, sample size, and limited observation time are the main limitations of this study. Additionally, definitions of success, failure, and complication are heterogenous within previous studies, rendering comparison difficult. Another limitation of this retrospective analysis represents the missing data concerning individual success rates of TLE based on lead type, fixation, manufacturer, or location. Complications were only assessed within 30 days after TLE, rendering assumptions on long-term complications impossible. 

## Figures and Tables

**Table 1:** Definitions. TLE: transvenous lead extraction.

Lead explantation	Removal of the lead by simple traction techniques and lead dwelling time below one year.
Lead extraction	Removal of at least one lead with a dwelling time of more than one year.
Complete radiological success	Removal of all targeted leads and material without any indwelling lead remaining.
Partial radiological success	Lead remnants <4 cm after TLE.
Radiological failure	Incapacity to remove lead, >4 cm of targeted lead remaining.
Clinical success	Achieving the clinical result for which the TLE was performed. Complete radiological success or partial radiological success, when the remaining lead fragment <4 cm, does not increase the risk of further complications, such as perforation, embolic events, conservation of infection, or any other undesired outcome. Absence of any procedure-related major complication resulting in permanent disability or death.
Clinical failure	Incapacity to achieve clinical success.
Major complications	All complications causing persistent or significant disability, life-threatening events or death, or necessitating surgical intervention to prevent the above.
Procedure-related major complications	Complications presumably related to TLE necessitating surgical intervention, requiring extension of hospitalization, causing persistent or notable disability, life-threatening events, or death.
Minor complications	Any undesired event occurring during the same hospital stay as TLE requiring medical intervention, observation, or minor procedural intervention without requiring extension of hospitalization, causing persistent or notable disability, life-threatening events, or death.
Reoperation	Surgical or interventional operation during the same hospital stay caused by a complication after TLE (procedure-related or unrelated).

**Table 2:** Patient, procedure, and lead characteristics.

Patients treated	350
Mean age (years)	63.6 ± 15.3
Male sex	251 (71.7%)
LVEF < 50%	168 (48.0%)
LVEF absolute (%)	45.7 ± 15.1
Hypertension	202 (57.7%)
Diabetes	73 (20.9%)
Renal insufficiency (eGFR < 60 mL/min)	121 (34.6%)
Anticoagulants Single antiplatelet therapy Dual antiplatelet therapy Oral anticoagulation Oral anticoagulation and antiplatelet therapy None	79 (22.6%) 6 (1.7%) 148 (42.3%) 16 (4.6%) 101 (28.9%)
Indication for TLE Lead dysfunction Infection Device upgrade Other	176 (50.3%) 108 (30.9%) 37 (10.6%) 29 (8.3%)
Procedure average duration (min)	131.7 ± 66.3
Mean lead dwell time (months)	112.5 ± 78.6
Type of lead Passive fixation ICD leads Dual-coil ICD leads Right ventricular leads Right atrial leads Left ventricular leads (coronary sinus)	115 (32.9%) 158 (45.1%) 76 (21.7%) 194 (55.4%) 243 (69.4%) 69 (19.7%).
Mean number of leads extracted Extracted leads per patient 1 2 3 4 5	1.9 ± 0.8 117 (33.4%) 165 (47.1%) 51 (14.6%) 12 (3.4%) 2 (0.6%)
Device reimplantation	263 (75.1%)

Specific patient and lead characteristics of this study population. Other indications for TLE include chronic pain, abandoned or recalled leads, and other lead-related complications. LVEF: left ventricular ejection fraction. eGFR: estimated glomerular filtration rate. TLE: transvenous lead extraction. ICD: implantable cardioverter–defibrillator. Plus–minus values are means ± SD. Percentages in relation to procedures performed.

**Table 3:** Major complications.

Type of Major Complication	Number of Affected Patients
Post-procedural multi-organ failure	6 (1.7%)
Procedure-related mortality	5 (1.4%)
Post-interventional cardiac decompensation	4 (1.1%)
Cardiac perforation requiring intervention	3 (0.9%)
Stroke	3 (0.9%)
Vascular perforation requiring intervention	2 (0.6%)
Post-procedural sepsis	2 (0.6%)
Hemothorax requiring intervention	1 (0.3%)
Anesthesiologic complication requiring intervention	1 (0.3%)
Plexus palsy	1 (0.3%)

Type of major complication on the left, absolute number of those complications, and percent of all 350 TLE (=100%) in brackets on the right. TLE: transvenous lead extraction.

**Table 4:** Mortality after transvenous lead extraction.

Specific Mortality	Number of Affected Patients
Procedure-related mortality	5 (1.4%)
In-hospital mortality	11 (3.1%)
All-cause mortality within 30 days	13 (3.7%)

Specific subgroups of mortality after transvenous lead extraction. Percentages are calculated from 350 performed extractions (=100%).

**Table 5:** Minor complications after transvenous lead extraction.

Type of Minor Complication	Number of Affected Patients
Hematoma without need for intervention	18 (5.1%)
Newly developed severe tricuspid regurgitation	10 (2.9%)
Swelling of the arm/thrombosis	10 (2.9%)
Pericardial effusion without need for intervention	8 (2.3%)
Blood transfusion	8 (2.3%)
Acute renal failure	7 (2%)
Vascular complications with minor intervention	6 (1.7%)
Hematoma with surgical revision	5 (1.4%)
Electrode dislocation requiring revision	4 (1.1%)
Pneumothorax requiring drainage	2 (0.6%)
Pulmonary embolism	2 (0.6%)
Anesthesiologic complication	2 (0.6%)

Type of minor complication on the left, absolute number of those complications, and percent of all 350 TLE (=100%) in brackets on the right. TLE: transvenous lead extraction.

## Data Availability

Upon urgent request and associated need, our data is available, while our utmost intention is to protect our patient’s privacy.

## Abbreviations

TLE	Transvenous lead extraction
CIED	Cardiac implantable electronic device
ICD	Implantable cardioverter–defibrillator
